# Absence without leave or leave without absence: Examining the interrelations among mind wandering, metacognition and cognitive control

**DOI:** 10.1371/journal.pone.0191639

**Published:** 2018-02-09

**Authors:** Leonhard Hakon Drescher, Eva Van den Bussche, Kobe Desender

**Affiliations:** 1 Department of Psychology, Vrije Universiteit Brussel, Brussels, Belgium; 2 Department of neurophysiology and pathophysiology, University Medical Center Hamburg-Eppendorf, Hamburg, Germany; 3 Department of Experimental Psychology, Ghent University, Ghent, Belgium; University of California, San Francisco, UNITED STATES

## Abstract

Despite the abundance of recent publications about mind wandering (i.e., off-task thought), its interconnection with metacognition and cognitive control has not yet been examined. In the current study, we hypothesized that these three constructs would show clear interrelations. Metacognitive capacity was predicted to correlate positively with cognitive control ability, which in turn was predicted to be positively related to resistance to mind wandering during sustained attention. Moreover, it was expected that participants with good metacognitive capacity would be better at the subjective recognition of behaviorally present mind wandering. Three tasks were used: The Sustained Attention to Response Task (SART) to measure mind wandering, a perceptual decision task with confidence ratings to measure metacognitive efficiency, and a conflict task to measure cognitive control. Structural Equation Modelling was used to test the interrelations among the three constructs. As expected, metacognitive efficiency was positively related to cognitive control ability. Surprisingly, there was a negative relation between metacognitive efficiency and the degree to which subjective mind wandering reports tracked the behavioral index of mind wandering. No relation was found between cognitive control and behavioral mind wandering. The results of the current work are the first to shed light on the interrelations among these three constructs.

## Introduction

The phenomenon of mind wandering, or cognitively veering away from the current external demands, is lately gaining attention in cognitive research and neuroscience [1–3]. It has long been ignored by researchers, despite its omnipresence among mental activities. Between 30% and 50% of our daily thought content is unrelated to our concurrent task, and can be classified as mind wandering (i.e., off-task thought; [4,5]). It is of vital importance to increase our understanding of mind wandering and its characteristics, because it can seriously hamper task performance, independent of task complexity (for a review, see [6]). Mind wandering has for example been shown to decrease performance on intelligence tests [7], and the memorization of university lecture content [8]. Furthermore, it has been linked to an increased risk of accidents while driving a car (for a review, see [9]). This detrimental effect on performance can be readily explained by the fact that mind wandering is accompanied by attentional disengagement from the concurrent (external) task or stimulus, also called perceptual decoupling [10,11]. This decoupling process does not follow an all-or-none rule: according to the levels-of-inattention hypothesis [12], perceptual decoupling follows the hierarchy of cognitive processing. In other words, there are slighter forms of mind wandering, where only higher-order processing decouples (i.e., weak decoupling), and profound forms of decoupling, when mind wandering affects low-level processing as well. Mind wandering can be measured by collecting subjective reports [10], but also through behavioral markers [13], enabling the differentiation between behavioral indices of mind wandering and subjective mind wandering reports.

An important feature of mind wandering is that while our minds wander, our level of consciousness is generally decreased. More specifically, mind wandering is characterized by the temporary absence of *meta-awareness* [11,14]. Meta-awareness is often described as the “explicit awareness of the contents of consciousness” ([15], p. 339). Theoretically, there is a strong overlap between this concept of meta-awareness and the concept of *metacognition*, which is typically defined as cognition about cognition [16,17]. Both concepts clearly refer to self-reflective processes, and the term metacognition can be used to encompass meta-awareness, among other similar constructs [18]. Metacognition is crucial for the realization that one’s mind is wandering [15], which implies that a relationship can be expected between metacognitive ability and how well someone can detect his/her own mind wandering. Participants with good metacognitive ability should be good at becoming aware that they are mind wandering (i.e., *mind wandering detection*), which might enable them to keep better focus on the task, counteracting the susceptibility to mind wandering. As a consequence, participants with good metacognitive ability can also be expected to display less behavioral mind wandering, assuming they are motivated to adhere to the task instructions. Indirect evidence for this proposed relation comes from studies indicating that mindfulness training, which is considered an exercise in meta-awareness, enhances metacognitive ability [19,20]. Interestingly, individual differences in a dispositional measure of mindfulness have also been found to be negatively correlated with mind wandering quantity [21,22].

Given that perceptual decoupling has been proposed as the underlying mechanism causing the detrimental effects of mind wandering on performance, mind wandering can be overcome by (re)focusing (i.e., recoupling) attention to the task. This refocusing requires *cognitive control*. Cognitive control is an umbrella term for a variety of processes, such as selective attention, response modulation and working memory, which together strategically adjust behavior for optimal performance [23]. Cognitive control is required to complete non-automated tasks, which are associated with low rates of mind wandering due to their high cognitive demand [10]. This implies that cognitive control processes may play an important role in the resistance against mind wandering, helping to regain task focus whenever the mind starts to wander. As a cautionary note, it should be mentioned that this relation is only expected if the mind wandering episode was not intentional. We therefore predict a negative relation between cognitive control ability and the quantity of behavioral mind wandering. Given that previous research has suggested that cognitive control relies crucially on metacognitive awareness [24], it can further be hypothesized that individuals with better metacognitive ability would also show better cognitive control ability.

**Fig 1:**
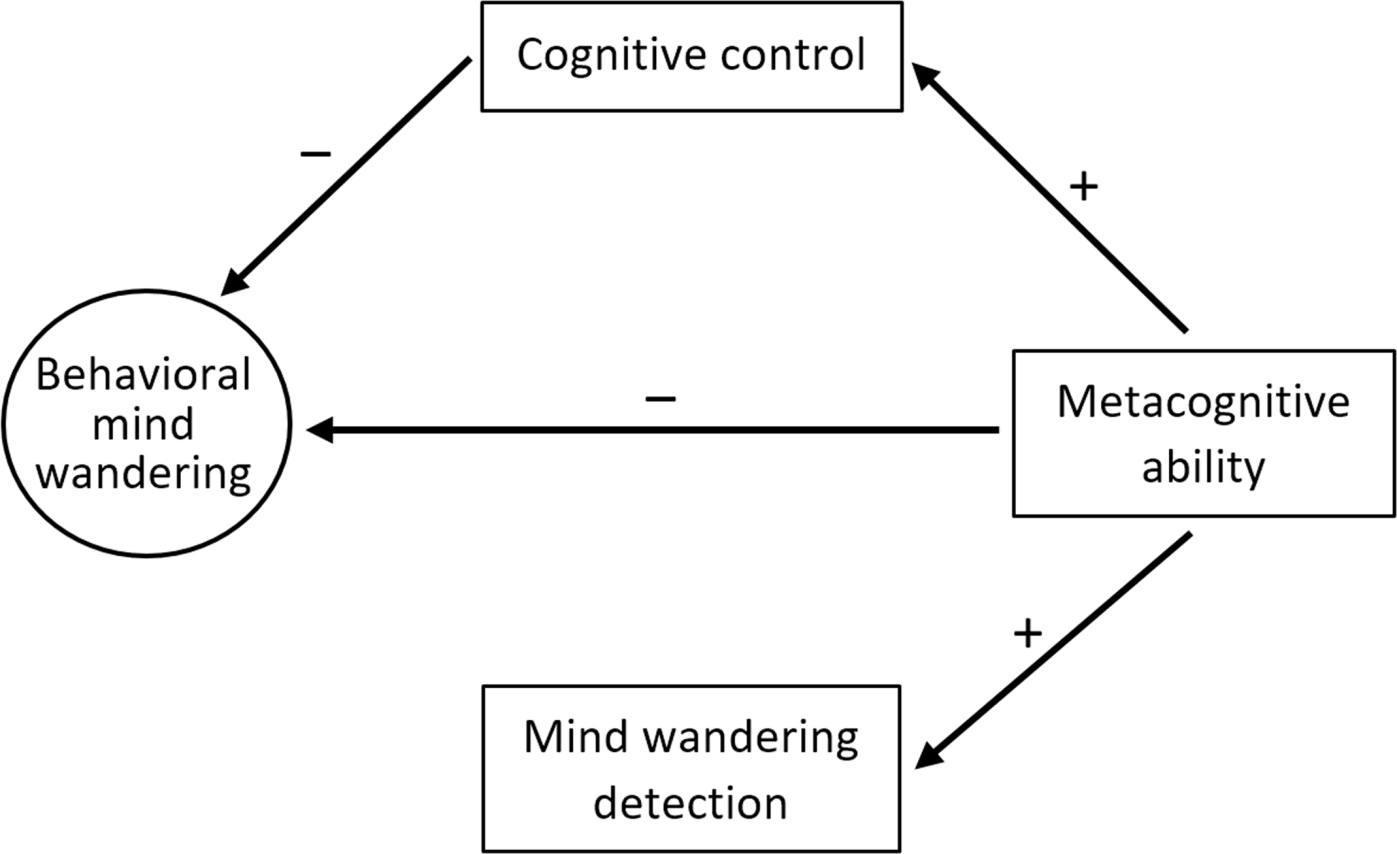
The predictions of the current experiment.

Based on the above it becomes clear that mind wandering, metacognition and cognitive control are likely interrelated constructs. Specifically, we predict that (a) better metacognitive skills are related to better subjective recognition of mind wandering episodes (i.e., mind wandering detection), that (b) better metacognitive skills are negatively related to the occurrence of mind wandering, that (c) better cognitive control is negatively related to the occurrence of mind wandering, and that (d) metacognitive skills are positively related to cognitive control. In order to test these assumed interrelations, participants performed three tasks that are known to reliably measure these three constructs. Mind wandering and mind wandering detection were measured using the Sustained Attention to Response Task (SART; [25]), metacognitive capacity was quantified by examining how well confidence ratings track performance in a perceptual decision task [26], and cognitive control was quantified by calculating the sequential modulation of the congruency effect (i.e., the Gratton effect; [27]), which is typically observed in conflict tasks where cognitive control is triggered. Fig 1 provides an overview of the expected interrelations.

## Method and materials

### Ethics statement

All experimental protocols were approved by the local ethics committee of the Vrije Universiteit Brussel (B.U.N. 143201422181). The experiments were performed in accordance with the relevant guidelines and regulations.

### Participants

In order to obtain sufficient statistical power (*α* = .05, 1 ‒ *β* = .80) to detect correlations of at least .35, we aimed for a sample of 61 participants. Power calculations were performed using the “pwr” library in R [28]. We were able to recruit 56 participants via the course credit program of the Vrije Universiteit Brussel. Since our pre-established sample size goal was not yet reached after this, eight additional participants were recruited in return for a remuneration of 10 €, adding to a total of 64 participants. All subjects provided written informed consent before the experiments began and were naïve with respect to the hypotheses. They also reported normal or corrected-to-normal vision. The data of one participant were removed from the analyses, because of low accuracy on the conflict task (< 80%). The final sample consisted of 63 subjects (50 female), aged between 17 and 24 years (*M*_age_ = 18.7 years, *SD* = 1.5).

### Procedure

All stimuli were presented visually on 15-in. CRT monitors with a vertical refresh rate of 85 Hz. Instructions were given in Dutch. Standard AZERTY keyboards were used to collect responses. To prevent order effects, the four parts of the experiment described below were partially counterbalanced in the following arrangements: ABCD, ABDC, BACD, and BADC. The two versions of the SART (i.e., C and D) were always presented last, in order to keep the circumstances constant under which mind wandering was measured. The total duration of the experiment was approximately 60 minutes.

### Sustained Attention to Response Task (SART)

**Fig 2:**
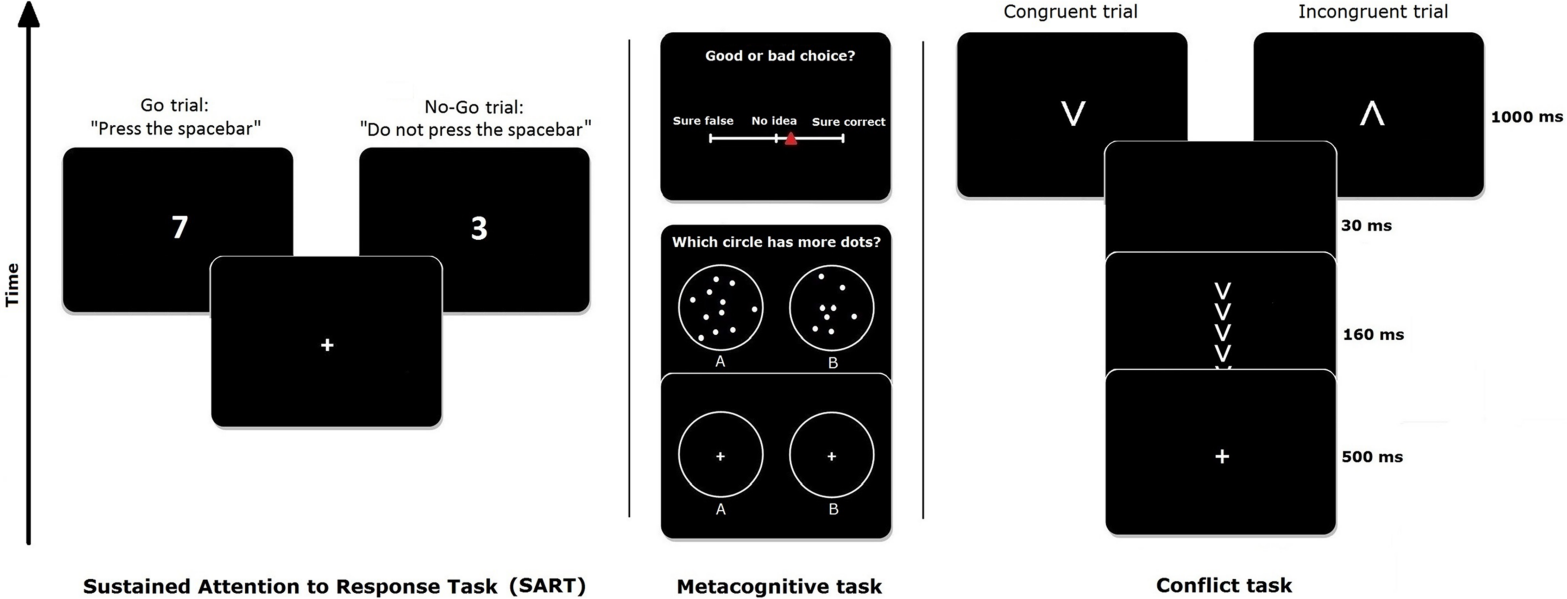
*Left*: The experimental paradigm of the SART. On each trial, a random digit (1–9) appeared on the screen, and subjects had to press the spacebar in response to all digits except 3. When the digit 3 appeared, they had to withhold their response (i.e., the No-Go trials). *Middle*: The metacognition task, adapted from Fleming et al. (2014). Two circles were presented with fixation crosses, followed by dot clouds (below). Participants had to choose the circle containing more dots. Subsequently, a rating of confidence was collected on a continuous bar with a red cursor (above). *Right*: The paradigm of the conflict task. Subjects were presented with a short prime stimulus consisting of a string of five arrows, pointing either in the same (congruent) or in the opposite (incongruent) direction as the subsequent target arrow. They were asked to respond to the direction of the target arrow.

Participants completed two versions of the SART, which were closely modelled to the original SART [25]. They were run on Inquisit [29], based on code provided online [30]. Stimuli were presented centrally on a black screen, consisting of single white digits (1–9), appearing for 300 ms in a quasi-random, pre-fixed order (i.e., the order was randomized a priori, but the same order was presented to each participant). After the digit stimulus, a mask appeared for 200 ms, which consisted of a circle (diameter 5.0°) with a diagonal cross inside. The mask was followed by a blank for 2000 ms. Digit font sizes (4.2°, 3.4°, 2.6°, 1.8°, and 1.1° vertically) were randomly varied in order to stimulate numerical processing [25]. Subjects had to perform a Go/No-Go task: they had to respond to the digits 1, 2, 4, 5, 6, 7, 8 and 9 (i.e., the non-target stimuli; Go trials) by pressing the space bar with the index finger of their dominant hand. They had to withhold this response when the digit 3 appeared (i.e., target stimulus; No Go trial). Targets appeared on 11% of the trials. Before completing the two versions of the SART, participants were given 20 practice trials with feedback about accuracy. See Fig 2 for an example of the SART trials. Prior to the experiment, subjects were instructed to keep their attention focused on the task, but they were also informed that off-task episodes may occur and should be reported accurately.

#### Probe-caught sampling

In the probe-caught version, participants performed 225 SART trials described above, during which 12 mind wandering probes were presented unexpectedly. The time interval between each probe semi-randomly varied between 30 to 90 seconds. When presented with a probe, participants indicated their cognitive state just before presentation of the probe, by selecting one of four options:

1. “I was fully focused on the task.”
2. “My mind was empty.” (i.e., empty minded samples)
3. “I thought about other things than the task, but I just noticed that now.” (i.e., unaware mind wandering samples)
4. “I thought about other things than the task and I was aware of it.” (i.e., aware mind wandering samples)

No time limit was imposed to respond to these probes. Subsequent to selecting an option, subjects were instructed to redirect their attention fully to the task, before the task continued. *Probe-caught subjective mind wandering* was inferred per participant from the number of selected response options 2 to 4 (i.e., mind wandering positive samples). Since in the current study the aim was to contrast off-task and on-task episodes, and in order to keep statistical power as high as possible, we did not differentiate between response options 2, 3 and 4 in the subsequent data analysis.

#### Self-caught sampling

In the self-caught version, subjects completed two blocks of 120 SART trials. They were asked to press the “B” key whenever they realized that their thoughts and attention had become unrelated to the task (i.e., when they detected that they were mind wandering). Pressing the “B” key immediately interrupted the task. A question screen with the following options was then presented:

1. “My mind was empty.” (i.e., empty minded samples)
2. “I thought about other things than the task.” (i.e., “classical” mind wandering samples)

The rest of the procedure was identical with the probe-caught version. *Self-caught subjective mind wandering* was inferred per participant from the number of “B” presses. For the same reasons as above, we did not differentiate between response options 1 and 2 in the subsequent data analysis.

### Metacognition task

To measure metacognition, participants performed a perceptual discrimination task combined with confidence ratings (see Fig 2), based on Fleming et al. [26]. The extent to which confidence ratings track the accuracy on the perceptual discrimination task has been found to be a good marker of metacognitive capacity [31]. Each trial began with the presentation of two white circles (5.1° in diameter) on a black background, arranged horizontally next to each other with a distance of 17.8° between the midpoints. Fixation crosses were shown for 1000 ms in each circle, followed by dot clouds in each circle that appeared for 700 ms. The dots had a diameter of 0.4°. Participants were requested to indicate the circle which contained more dots by pressing either the “S” or the “L” key with the index fingers of either hand. Subsequently, the question “Good or bad choice?” appeared, with a continuous confidence rating bar, ranging from “Sure false” to “Sure correct”, with “No idea” as a middle point. Participants were told to move a cursor on the confidence rating bar with the “S” and “L” keys (the “S” key moving the cursor to the left, and the “L” key moving it to the right), and to confirm their confidence judgment by pressing the enter key. No time limit was imposed for both the primary choice and the confidence rating.

The difference in the number of dots appearing in the two circles (i.e., the difficulty of the task) was adapted dependent on the participant’s performance, keeping the accuracy rate at approximately the same level for all participants. This was achieved by changing the amount of dots according to an unequal step size staircase procedure, which yields good convergence to perceptual discrimination thresholds [32]. Specifically, the difference in dots was increased by two dots if the participant’s answer was incorrect, and decreased by one dot if the answer was correct. After four consecutive reversals between correct or incorrect responses, the step size was doubled (four dots and two dots respectively), and after eight consecutive reversals the step size returned to the original two dots and one dot. This procedure led to an average accuracy of 74.8% (*SD* = 1.5%). Dot positions in the boxes, as well as the position of the box containing more dots (left or right) were randomly selected on each trial. Subjects first received several practice trials (10 without confidence rating, 14 with confidence rating), before they completed eight experimental blocks of 25 trials.

### Conflict task

To acquire an index of cognitive control, participants performed a conflict task [23,33]. Here, we used an arrow priming task, in which participants have to decide as fast as possible on the direction of a target arrow. This target arrow is always preceded by a prime arrow which can either trigger the same response (i.e., a congruent trial) or a different response (i.e., an incongruent trial) than the target [34]. Reaction times (RTs) are reliably longer on incongruent trials compared to congruent trials, which is known as the congruency effect [34]. This effect is also influenced by the congruency of the previous trial. More specifically, the congruency effect is decreased following an incongruent trial compared to a congruent trial, a phenomenon known as the Gratton effect [27]. This Gratton effect is interpreted as a manifestation of *reactive* cognitive control [23]: after an incongruent trial, the focus on the relevant features of the task (i.e., the target) is enhanced leading to a reduced congruency effect on the subsequent trial.

Primes and targets consisted of white V-shaped arrows against a black background. The five prime arrows (size 1.4° each) were randomly arranged in either a vertical or a horizontal string, all pointing to either side of the string axis (up, down, right, or left). The single target arrow (size 2.0°) appeared in the middle of the screen, pointing in the same direction as the primes (congruent condition), or in the opposite direction (incongruent condition). Each trial began with a fixation cross in the middle of the screen for 500 ms, followed by the primes for 160 ms. Then, a blank screen was inserted for 30 ms, before the target was presented for 1000 ms (see Fig 2). Subjects were instructed to place the index and middle finger of their left hand on the “D” and “F” keys respectively, and the same fingers of their right hand on “J” and “N”. Responses were given according to the target direction: the “D” and “F” keys corresponded with left and right, and the “J” and “N” keys corresponded with up and down. After 12 practice trials with feedback (i.e., the message “correct” or “false”), participants were requested to complete 160 trials, divided into four blocks of 40 trials. Each block contained an equal number of congruent and incongruent trials. Consecutive trials always switched from the horizontal to the vertical dimension, eliminating feature repetitions and memory confounds [34]. The task was created and run in E-prime 2.0 [35].

### Data analysis

#### Mind wandering

In the literature on mind wandering, different response variables have been proposed as *behavioral markers* of mind wandering in the context of the SART paradigm, which was used in the current study. Proposed indices include the percentage of failure on No-go trials, the percentage of failure on Go trials, the variation in RTs on Go trials, and the percentage of trials with anticipatory RTs on Go trials (i.e., RTs < 100 ms) [13]. A drawback of having many response variables of the same underlying phenomenon is that each variable by itself probably only captures a limited aspect of behavioral mind wandering. Moreover, having several indicators of the same underlying phenomenon comes with the risk of cherry-picking the indicator that seems to “work” [36]. Therefore, in the current study we constructed an integrated index of behavioral mind wandering using a data driven approach. Specifically, using factor analyis, a single latent variable was extracted from the four known behavioral indicators of mind wandering. This was done separately for the probe-caught and self-caught versions of the SART. The calculation proved to be a successful approach to behavioral mind wandering (see below).

In addition, we calculated a subjective *mind wandering detection* index for each participant. The rationale for this calculation was that the values on our behavioral mind wandering index should be high when participants indicate that they are mind wandering, whereas these values should be low when participants do not report that their mind is wandering. The better participants’ subjective mind wandering reports match this behavioral factor (low factor for on-task; high factor for off-task), the better they are at subjectively detecting mind wandering episodes. To calculate mind wandering detection indexes, the subjective reports, which were classified as on-task and off-task reports, were taken as reference points. Factor analyses were computed on the trials *preceding* each subjective report, seperately for off-task and on-task reports. Due to the difference in design, the exact method depended on the version of the SART:

In the probe-caught version of the SART, all segments of four trials preceding the on-task reports (answer option 1) were pooled together for the calculation of an on-task latent factor. Also, all segments preceding the off-task reports (answer options 2 to 4) were pooled together to extract an off-task latent factor. This was done separately for each participant. We also calculated this index while excluding the behavioral data preceding response option 4 (i.e., aware mind wandering) from the off-task factor. This was done in order to examine whether aware mind wandering confounded the detection index. However, this calculation led to a severe reduction in data points and hence statistical power. Notwithstanding, the resulting detection index correlated highly with the original index, *r*(52) = .97, *p* < .001, indicating that both measure the same construct.

In the self-caught version, the off-task latent factor was extracted from the pooled segments of four trials leading up to each self-initiated report of mind wandering (off-task reports). This factor was then compared to the on-task latent factor extracted based on all remaining trials, which were considered on-task trials. This was also done separately for each participant.

By means of this calculation we obtained off-task and on-task factor values for each participant and each version of the SART. Within each version, the difference between the two factor values (off-task–on-task) was then taken as an index of the ability to detect mind wandering. A large difference between on-task and off-task factor values indicates that participants’ subjective and behavioral data are a good match between subjective and behavioral data (i.e., good mind wandering detection), whereas a small difference indicates that there is a poor match between both (i.e., poor mind wandering detection). Note that it is also technically possible that there are no differences in performance between on-task and off-task performance to start with, which would then result in a zero difference on our subjective mind wandering detection index. However, given that a large body of evidence suggests that episodes of mind wandering are accompanied by decreases in performance [13,37], we believe this alternative to be rather unlikely. Provided that subjects are motivated to perform the task well, the behavioral factor should reflect whether a participant is mind wandering or not, and the mind wandering detection index should be high (i.e., high difference between on-task and off-task factors) if participants’ subjective reports match the behavioral factor.

#### Metacognition

Based on the data from the metacognition task, we calculated the recently developed meta-*d’* measure per participant [38]. Meta-*d’* is an indicator of *metacognitive sensitivity*, derived from signal detection theory. It is free from metacognitive bias (i.e., an overall tendency to show high or low confidence ratings), and from differences in performance on the primary task (in our case, performance on the dot discrimination task), also termed type 1 performance *d’* [38]. The type 1 performance *d’* is expressed on the same scale as meta-*d’*. By then computing the meta-*d’*/*d’* ratio, it is possible to quantify metacognitive capacity, which indexes how much first order information is used for the construction of a confidence judgment. When the ratio meta*-d’*/*d’* is equal to 1, this indicates that *all* first-order information is used when providing a confidence rating. When this ratio is smaller than 1, participants do not use all first-order information when providing a confidence judgment (i.e., they are metacognitively inefficient).

#### Structural Equation Modelling (SEM)

To test the interrelations between our indices of mind wandering (behavioral mind wandering and mind wandering detection), metacognitive efficiency, and cognitive control, a structural equation model (SEM) was fit to the data. The R package “mvoutlier” [39] was used, which can detect outliers when there are multiple variables. More specifically, we applied the adjusted quantile plot function “aq.plot” to our data using default values, namely a chisq quantile of .975, a delta of .05, and an alpha of .05 (see [39]). We found there to be 25 outliers in the probe-caught version (out of *n* = 61), and 13 outliers in the self-caught version (out of *n* = 63). The raw values of each variable are depicted in S1 File. Given the large number of outliers, classical linear regression within the SEM was not an appropriate approach to analyse the data, and excluding these outliers from the data would have severely reduced the statistical power. Instead, we opted for robust regression, a technique which aims to increase the reliability of statistical modeling through reduced sensitivity for extreme observations. Importantly, this way the model can be fit to the data of *all* participants (i.e., without excluding participants exceeding some arbitrary cut-off value), while downweighting the influence of outliers. In S1 Text, we further demonstrate that robust regression provides a better fit to the data than classical linear regression, and we additionally report the results with classical regression on the data of all participants and the data after excluding outliers. The robust regressions reported in the results section are based on MLM-estimation, which uses a maximum likelihood estimator with robust standard errors and test statistics [40,41]. Model fit was assessed with three different indices: (1) The chi-square statistic testing the difference between observed and reproduced covariances, for which a non-significant outcome indicates that most of the observed covariance between variables can be explained by the model. (2) The Comparative Fit Index (CFI), indicating the difference of the fitted model from the baseline null model. CFI values larger than .90 are indicative of adequate model fit [42]. (3) The Root Mean Square Error of Approximation (RMSEA), which is based on the population error of approximation. RMSEA values below .05 indicate close fit [43], while a value between .05 and .08 represents a fair fit, and a mediocre fit with a value of .08 to .10 [44].

#### Statistical computations

The metacognition task was run on Psychophysics Toolbox version 3 [45] in Matlab 2010a, based on code provided online [46]. The results thereof were calculated in Matlab 2010a with additional code retrieved online [38]. All of the subsequent statistical analyses were carried out in SPSS 20.0 and R 3.3.1 [47]. For Structural Equation Modelling, the package “lavaan” (version 3.3.2) was used [48].

## Results

### Mind wandering

The amount of subjective mind wandering reports were roughly the same within the two versions of the SART: Participants reported that they were, on average, mind wandering 7.3 times during the self-caught version (*SD* = 5.8), and out of the 12 probe-caught samples, 5.9 were classified as mind wandering positive (*SD* = 2.7). Most of the self-caught samples were “classic” mind wandering (*M* = 70.6%, *SD* = 23.4%), the rest were empty minded samples (*M* = 29.4%, *SD* = 23.4%). Of the mind wandering positive samples on the probe-caught version, the largest proportion were aware mind wandering (*M* = 23.8%, *SD* = 15.0%), and a smaller proportion were unaware mind wandering (*M* = 9.9%, *SD* = 10.5%). The remainder were empty minded probe samples (*M* = 15.1%, *SD* = 13.3%). These proportions are comparable to previous experiments that have used the SART [3]. The number of positively answered probe-caught samples correlated with the number of self-caught samples, *r*(61) = .34, *p* = .006, and both factors of behavioral mind wandering (self-caught and probe-caught) were also correlated, *r*(61) = .88, *p* < .001, suggesting good internal consistency.

As explained above, we then computed two indices of behavioral mind wandering (i.e., probe-caught and self-caught) and two indices of mind wandering detection (i.e., probe-caught and self-caught). These values were used in the between-task analyses reported below. The results indicated that our novel approach to construct a single factor of behavioral mind wandering was appropriate, given that all four individual indices of behavioral mind wandering showed moderate to high factor loadings (between .52 and .91; see below) on the latent factor of behavioral mind wandering, which indicates that this factor analytic approach was successful to quantify the occurrence of behavioral mind wandering.

### Metacognitive efficiency

**Fig 3:**
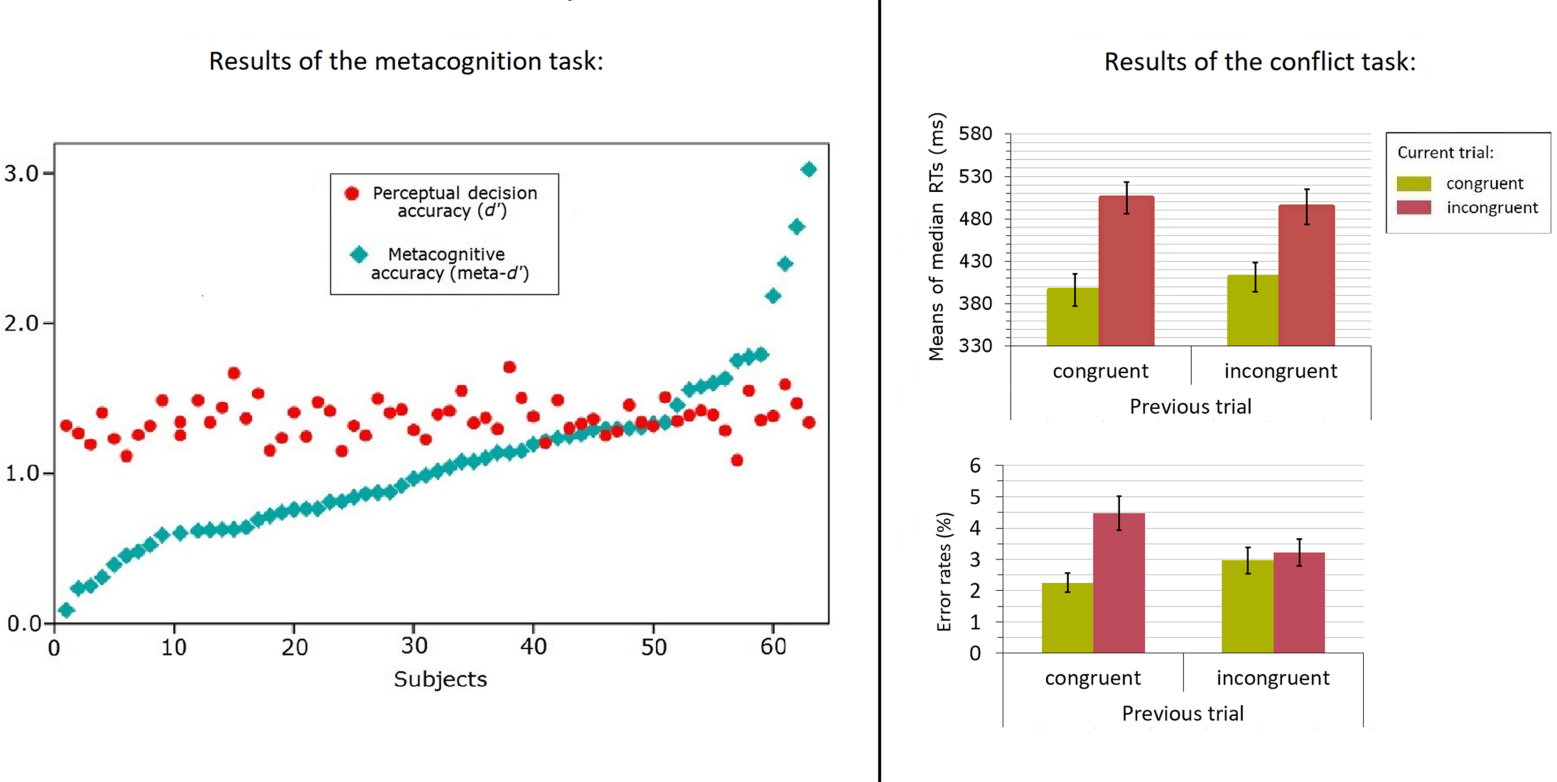
*Left*: Scatterplot of metacognitive accuracy (meta-*d’*; blue diamonds), and primary task performance (*d’*; red dots). The data were arranged by augmenting meta-*d’* values. *Right*: 2×2 factorial plots of the conflict task, separately for the means of the median RTs (above), and the means of the error rates (below), for each condition defined by congruency and previous congruency. Error bars reflect the 95% confidence intervals for the means.

Replicating previous work, meta*-d’* showed considerable variability across participants (*M* = 1.07, *SD* = .56), whereas type 1 performance (*d’*) did not show much variation (*M* = 1.36, *SD* = .13; see Fig 3). The meta*-d’*/*d’* ratio was used as an indicator of metacognitive efficiency in the between-task analyses reported below. The mean meta*-d’*/*d’* ratio was 0.78 (*SD* = .40).

As can be seen in Fig 3, several participants had meta*-d’* values that were higher than their corresponding *d’* value, indicating they used more information in their metacognitive judgment than in their primary judgment. Although this might seem puzzling, it can be explained by the notion that evidence continues to accumulate after the primary response is given [49]. This information is not yet available during the primary response (e.g., it is still in the processing pipeline; [50]), but it can be used when providing a confidence judgment. This mechanism can explain why some people achieve a higher meta-*d’* than *d’*. If correct, this hypothesis predicts that cases where meta-*d’* is larger than *d’* should primarily be observed when RTs on the primary task are fast (i.e., making it more likely that there is still evidence in the processing pipeline). Confirming this hypothesis, we found that median RTs on the metacognition task were negatively correlated with meta-*d’*/*d’*, *r* = -.39 *p* = .001, indicating that participants whose primary responses were, on average, rather fast, were more likely to display higher sensitivity in their confidence judgments than in their primary task performance. In the results section, we discuss the implications of this additional result for the validity of the metacognitive index.

### Cognitive control

The data of the conflict task were used to calculate an index of cognitive control per subject. The first trial of each block (2.4% of the total trials), error trials (3.3%), and trials following an error (3.6%) were removed from the analysis. Median RTs on correct trials and mean error rates were submitted to a repeated measures ANOVA with congruency (congruent vs. incongruent) and previous congruency (congruent vs. incongruent) as independent variables. A main effect of congruency was found, *F*(1, 62) = 246.32, *p* < .001, *η*_*p*_^2^ = .80, showing that RTs were faster on congruent (455 ms) compared to incongruent trials (535 ms). There was also a significant interaction between congruency and previous congruency, *F*(1, 62) = 12.23, *p* = .001, *η*_*p*_^2^ = .17. As predicted, congruency effects were markedly reduced when the previous trial was incongruent (70 ms) compared to when it was congruent (90 ms, i.e., the Gratton effect; see Fig 3). The main effect of previous congruency was not significant, *F*(1, 62) = 1.75, *p* = .19, *η*_*p*_^2^ = .03.

The results of the error rates replicated this pattern: there was a significant main effect of congruency, *F*(1, 62) = 7.09, *p* = .01, *η*_*p*_^2^ = .10, showing that incongruent trials lead to more errors (3.85%) than congruent trials (2.61%). The interaction between congruency and previous congruency was also found in the error rates, *F*(1, 62) = 8.68, *p* = .005, *η*_*p*_^2^ = .12. Similar to the RT results, congruency effects were markedly reduced when the previous trial was incongruent (0.25%) compared to when it was congruent (2.23%, i.e., the Gratton effect). There was no significant main effect of previous congruency, *F*(1, 62) = 0.54, *p* = .47, *η*_*p*_^2^ = .009.

To obtain an index of *cognitive control* per participant, we calculated the Gratton effect for each participant by subtracting the RT congruency effect following incongruent trials from the RT congruency effect following congruent trials. This index is used in the between-task analyses reported below.

### Between-task results (SEM)

**Fig 4:**
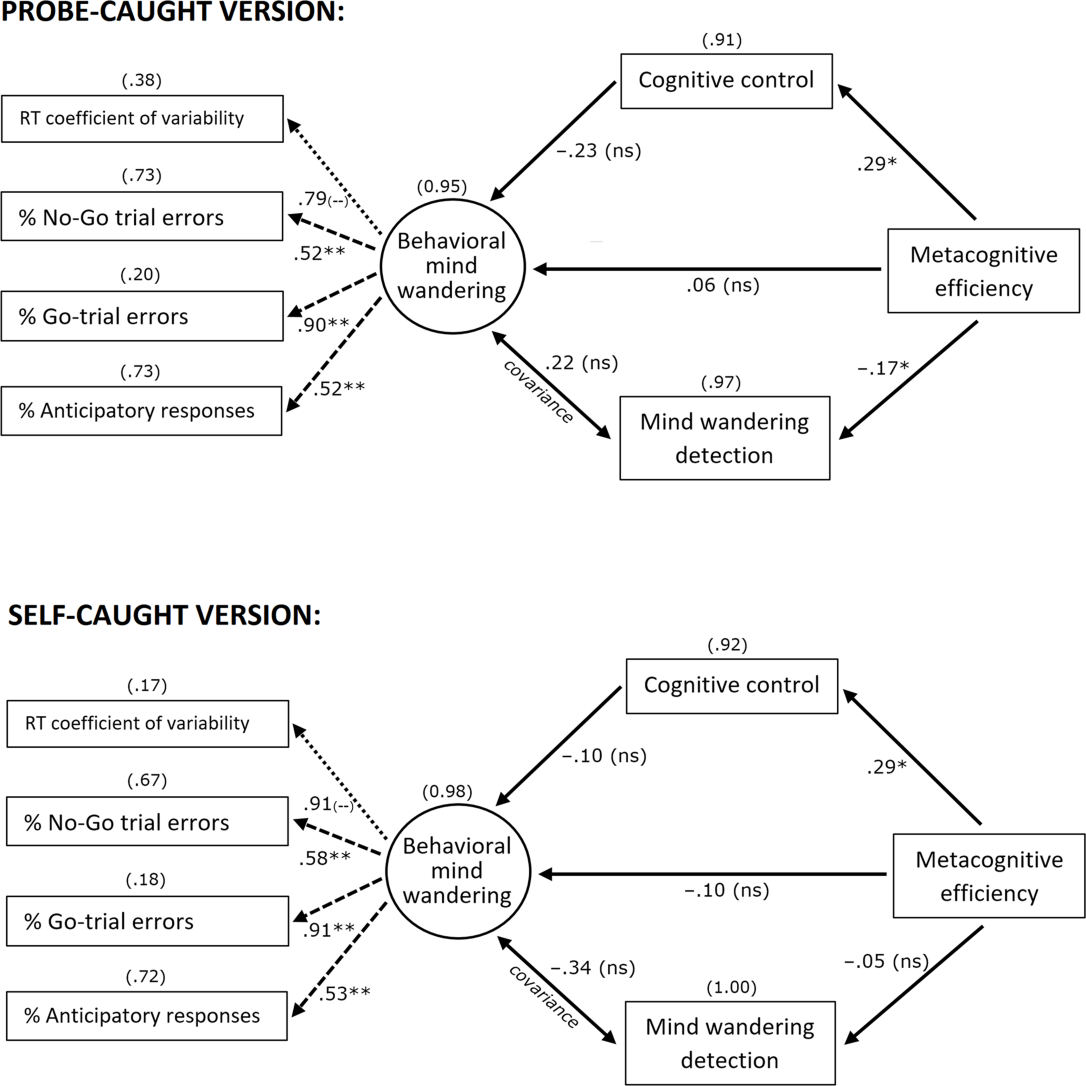
The structural equation model for each version of the SART. Standardized estimate values are shown for each regression, and the residual covariance. Variances are displayed in brackets above each dependent variable. * *p* < .05, ** *p* < .01, (--) significance value was not calculated.

Because of missing data in the probe-caught version of the mind wandering detection index, two subjects had to be excluded from further analysis. The probe-caught SEM was thus calculated from the data of 61 participants, and the self-caught SEM from the original sample of 63 participants. The model fit was good in both versions of the SEM: in the probe-caught version, *χ*^*2*^ (12) = 12.98, *p* = .37, CFI = .97, RMSEA = .037, and in the self-caught version, *χ*^*2*^ (12) = 13.49, *p* = .33, CFI = .96, RMSEA = .044. The SEM models and the regression values within the models are depicted in Fig 4.

As expected, metacognitive efficiency was positively related to cognitive control, both in the probe-caught version, *β* = .29, *p* = .04, and in the self-caught version, *β* = .29, *p* = .04. This indicates that participants with better metacognitive accuracy also had better cognitive control ability. Contrary to our expectation, a *negative* relation was found between metacognitive efficiency and mind wandering detection in the probe-caught version, *β* = ‒.17, *p* = .02. This relation was absent in the self-caught version, *β* = ‒.05, *p* = .62. The hypothesis that metacognitive efficiency would be positively related to the presence of behavioral mind wandering was disconfirmed, both in the probe-caught version, *β* = .06, *p* = .66, and in the self-caught version, *β* = ‒.10, *p* = .46. Our hypothesis that cognitive control would show a negative relation with behavioral mind wandering was also disconfirmed, in the probe-caught version, *β* = ‒.23, *p* = .32, and in the self-caught version, *β* = ‒.10, *p* = .53 (although the linear tendencies were as expected). Finally, the covariance between behavioral mind wandering and mind wandering detection was not significant, neither in the probe-caught version, *β* = .22, *p* = .40, nor in the self-caught version, *β* = ‒.34, *p* = .27.

## Discussion

The goal of the present study was to examine how mind wandering, metacognition and cognitive control are related to one another. The results of all individual tasks replicated the findings from prior research that they were modelled after. A novel approach of the current work was that different behavioral markers of mind wandering were used to construct one latent factor of behavioral mind wandering. This approach succesfully dealth with the problem of selecting an appropriate indicator of behavioral mind wandering among several potential reponse variables. Results of a structural equation model fit showed a significant positive relation between metacognitive efficiency and cognitive control. The better participants’ confidence judgments tracked their perceptual decision accuracy, the larger they reactively increased control in a conflict task. Furthermore, we observed an unexpected negative relation between metacognitive efficiency and the subjective detection of behaviorally present mind wandering. Interestingly, our latent variable of behavioral mind wandering was unrelated to both metacognitive efficiency and cognitive control. In the remainder, we discuss possible interpretations of these findings, and provide suggestions for future research that can increase our understanding of the relation between mind wandering, metacognition and cognitive control.

### Reliability of the individual tasks

#### SART

The results of the SART showed that both versions of the task (self-caught versus probe-caught) correlated on behavioral markers and subjective sampling, suggesting good internal consistency despite essentially differential sampling methods. One exemption is the aforementioned observation that the percentage of errors committed on target trials (i.e., the No-Go trials) showed high factor loadings when it was used to construct our detection index, except for the off-task factor of the self-caught version, where it had a small factor loading. One explanation for this unexpected finding might be that in the self-caught version, target errors are interpreted by participants as an indication that they are mind wandering, which does not necessarily have to be the case. This can explain why this variable was not a good predictor of behavioral mind wandering, specifically in this particular version. Apart from this exception, however, our approach to construct a single behavioral indicator for off-task thought—which has not yet been established in literature—proved to be successful in obtaining a single indicator of behavioral mind wandering.

#### Metacognition task

The results of the metacognition task were comparable to the study it was modelled after [26]. Similar levels of type 1 accuracy were obtained, indicating the successful application of the unequal step size method used for updating the dot contrast between the circles. Metacognitive efficiency showed strong variability across participants, which has been interpreted in the literature as differences in metacognitive ability.

#### Conflict task

The results of the conflict task showed a stable Gratton effect, replicating the findings by Schmidt and Weissman [34], although effect sizes were smaller than in their results. We thus obtained a reliable measure of cognitive control, which was free from feature repetitions or memory confounds [34].

### Main hypotheses

We conjectured that metacognition should be negatively linked to behavioral mind wandering. This assumption was motivated by the idea that metacognition should be related to cognitive control. Put simply, we predicted participants with better metacognition to have better cognitive control, and thus to be better in suppressing off-task thought. We did indeed observe a positive relation between metacognition and cognitive control. Participants who were better at evaluating their own accuracy showed larger reactive control in reaction to response conflict. This observation is in line with our previous work, where it was suggested that cognitive control critically depends on metacognitive awareness [24]. The critical novelty of the finding in the current work, is that we were able to show that individual differences in metacognitive efficiency are related to individual differences in cognitive control. Our hypothesis that metacognition would also be related to behavioral mind wandering was, however, not confirmed by the data.

We further predicted that differences in metacognitive efficiency would be related to the subjective detection of behaviorally present mind wandering. Participants who are better at detecting their own errors should also display more accurate reporting of mind wandering. Indeed, in the probe-caught version, we did observe a relation between both variables. Interestingly, however, this relation was opposite to what was predicted: participants with good metacognitive efficiency were more likely to be *worse* at detecting their own mind wandering episodes. A clear explanation for this finding is lacking, however in the section below on *issues regarding construct validity*, we bring forward some explanatory interpretations. Interestingly, this negative relation suggests that differences in movation cannot account for the (lack of) relations observed in the current work, because clear differences in motivation between participants would result in a positive relation between both variables.

As for our final hypothesis, we found no relation between cognitive control and behavioral mind wandering. Note that a positive relation between both variables is only expected when participants refocus their attention to the task once they notice that their thoughts have gone off-task. If participants lack the motivation to do so (e.g., in cases of intentional mind wandering; [51]), this prediction is, however, no longer accuracte. Therefore, future studies attempting to unravel the relation between mind wandering and related theoretical constructs should take the occurrence of intentional mind wandering into account.

### Assessment of construct validity

Although the results of the individual tasks that were used in the current study were highly comparable to that of previous work, some of the predicted relationships were not observed. Beside the possibility that these theoretically related constructs are actually not related, it could also be that the tasks we used did not validly measure the constructs they were supposed to measure.

#### Validity of the mind wandering detection index

The mind wandering detection index might be confounded by the presence of intentional mind wandering. It has recently been found that motivation and intention are important factors for mind wandering frequency [51]. Following this intention-dependent account, participants with good metacognitive efficiency may have frequent episodes of intentional mind wandering which they do not report, and as a consequence it might seem as if they are actually worse at detecting their own mind wandering. This might explain the unexpected negative relation between metacognitive efficiency and our subjective mind wandering detection index. Another problem with the detection index, that might explain this unexpected finding, is based on the hypothesis that perceptual decoupling occurs in graded levels [12]: It could be that subjects with higher metacognitive efficiency are more resistent to profound decoupling. This would imply that they had a larger proportion of slight decoupling (i.e., higher-order processing decouples but lower-order processing remains coupled). If this is true, partial decoupling would be reflected in the principal component values, but would not be reported. Hence, in participants with higher levels of metacognitive accuracy, the off-task factor would not be higher than the on-task factor, indicating poor mind wandering detection. The failure to report such partial decoupling might be due to the fact that enough attention remains coupled to the task, not classifying it as an off-task episode for the individual. This could be the case during cognitions such as task related interference, when the participant engages in internal cognitions which are about the task and the participant’s performance of the task [52]. The complete absence of a significant relationship between metacognitive efficiency and mind wandering detection in the self-caught version may be because participants were reminded by their target errors that they had just been mind wandering, rather than by their own thought monitoring. It might be that following a target error participants would report mind wandering, because having made an obvious error may entail the assumption that one’s mind has been off-task. The latter would also explain why participants did sometimes report being empty minded in the self-caught version, rather than solely reporting full mind wandering.

#### Validity of the metacognitive index

In the current work, individual differences in metacognitive efficiency were quantified by how well participants’ confidence ratings tracked their actual performance. Specifically, we calculated meta-*d’*/*d’* which is typically taken to be an index of *metacognitive efficiency*. A potential problem with this measure, however, might be that it is susceptible to differences in motivation between participants. Specifically, participants are not rewarded to accurately report their confidence, and might thus lack the motivation to do so (for a discussion on this, see [53]). Interestingly, we observed that meta-*d’*/*d’* values were negatively correlated to overall RTs on the perceptual decision task, showing that subjects who responded faster on the perceptual decision trials tended to give more accurate confidence judgments. This finding can be interpreted based on Pleskac and Busemeyer [49], who proposed that evidence continues to accumulate after the primary response. Although this information is not yet available during the primary response (e.g., it is still in the processing pipeline; [50]), it can be used when providing a confidence judgment. This post-evidence accumulation mechanism can explain why some people achieve a higher meta*-d’* than *d’*. An alternative interpretation of this negative correlation is that participants who were highly motivated responded more quickly and also put more effort into providing accurate confidence ratings (which would lead to a higher meta-*d’/d’* ratio). Whatever the correct explanation for this correlation, it raises the problematic possibility that the meta*-d’/d’* ratio is not a pure measure of metacognitive efficiency. Even though this measure of metacognitive efficiency has been successfully adopted in previous work [20,54], its lack of a correlation with theoretically related constructs as well as its correlation with overall RTs, calls for a further in-depth evaluation of its usefulness as an index of metacognitive efficiency.

#### Validity of the cognitive control index

Finally, it should be noted that the Gratton effect, our index of cognitive control, does only measure one specific aspect of cognitive control, namely *reactive* cognitive control to response conflict. The conflict adaptation we measured with the control task is comparable to the response inhibition needed on No-Go trials of the SART. However, performance on the SART also relies on sustained attention, which involves the activity of other networks than response inhibition [55]. Sustained attention relies predominantly on *proactive* control, which is characterized as an active maintenance of attention, anticipating cognitively demanding events [56]. In contrast, the conflict paradigm we used measures *reactive* control, or the adaptation to conflict after its occurrence. Hence, even though reactive control clearly plays a role in refocussing to the task, it does not appear to have an impact on mind wandering frequency. Future reseach should therefore test the impact of proactive control ability on mind wandering frequency, in the search to unveil the precursory factors for mind wandering alltogether.

### Conclusion

In the current work, we examined the relation between mind wandering, metacognition and cognitive control. We used a latent variable approach to quantifiy behavioral mind wandering based on several known response variables indicating mind wandering on the SART. The data showed a positive relation between the measures used for metacognition and cognitive control, as well as a negative relation between the measures used for metacognition and the subjective detection of mind wandering. Unexpectedly, there were no relations between our indexes for behavioral mind wandering and metacognition nor for behavioral mind wandering and cognitive control.

## Supporting information

S1 File Raw value distribution graphs of all variables included in the SEMs. (PDF)

S1 Text Comparison of the outcome values of the SEMs. (DOCX)
