# Supplementary material for: Absence without leave or leave without absence: Examining the interrelations among mind wandering, metacognition and cognitive control
Source: PLoS One. 2018 Feb 9;13(2):e0191639. doi: 10.1371/journal.pone.0191639 (PMC5807058; doi:10.1371/journal.pone.0191639)

S1 File.
Raw value distribution graphs of all variables included in the SEMs.

SART RESPONSE VARIABLES: PROBE-CAUGHT VERSION

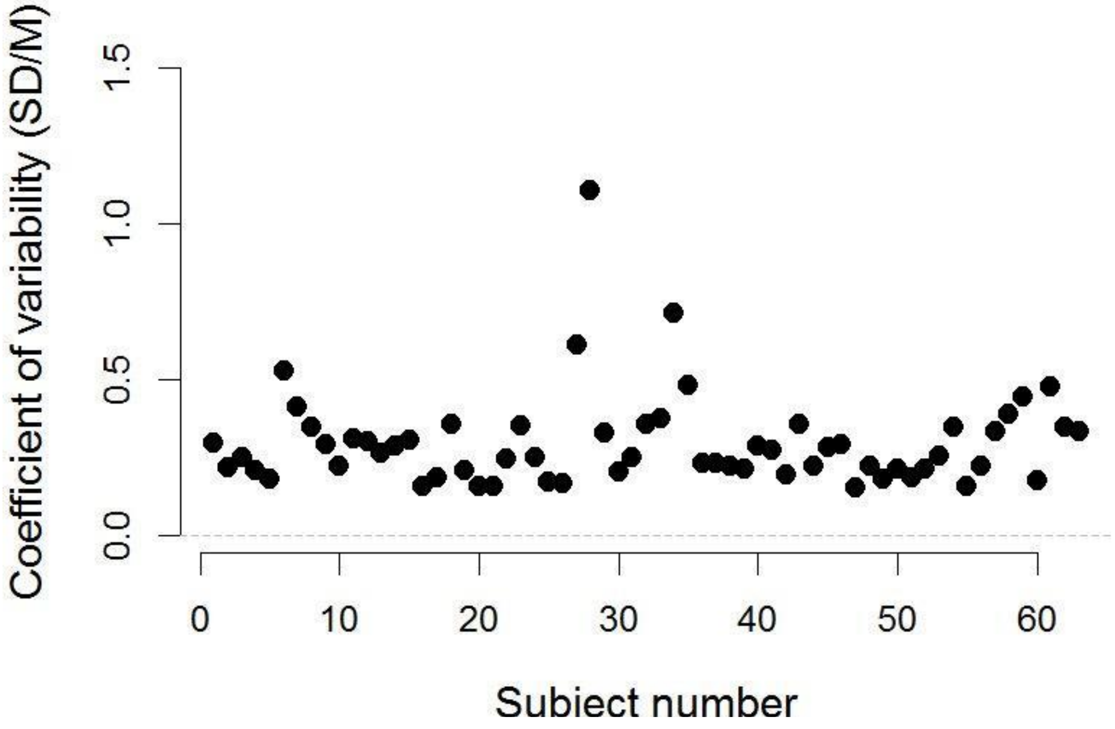

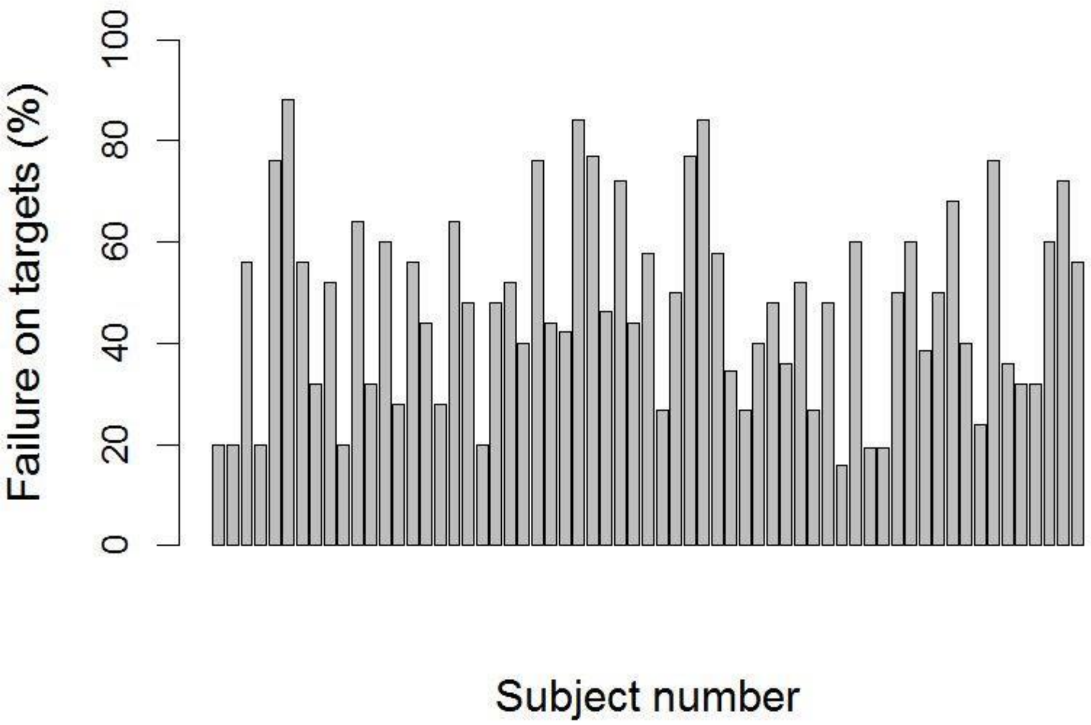

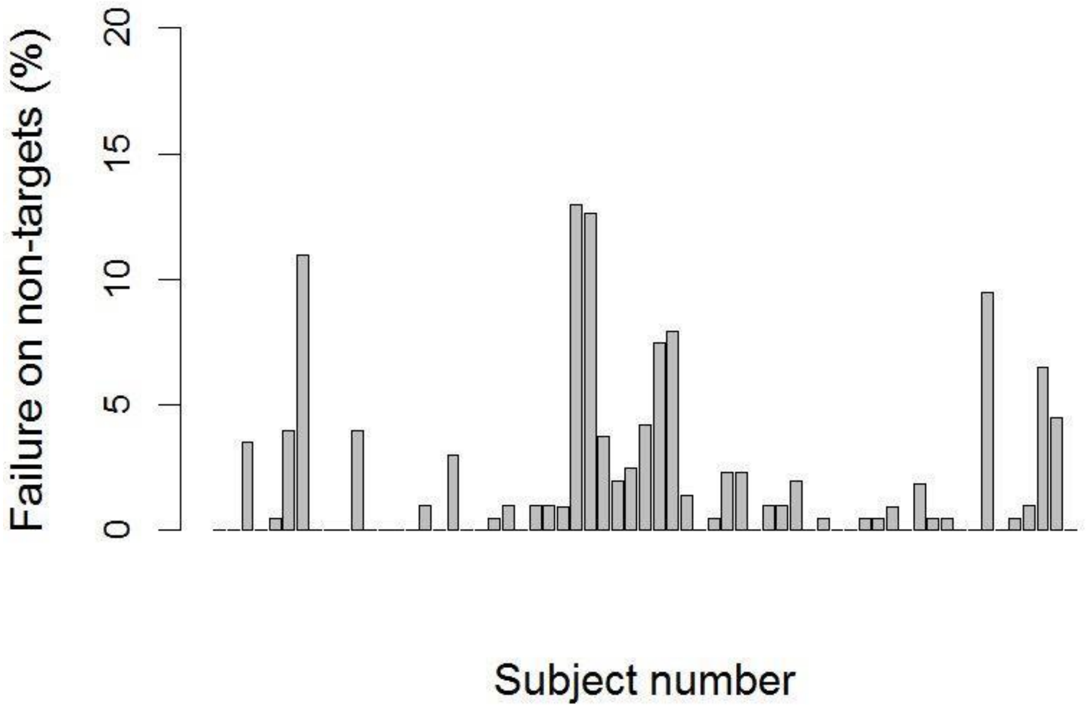
Failure on non-targets (%)
0
5
10
15
20
Subject number

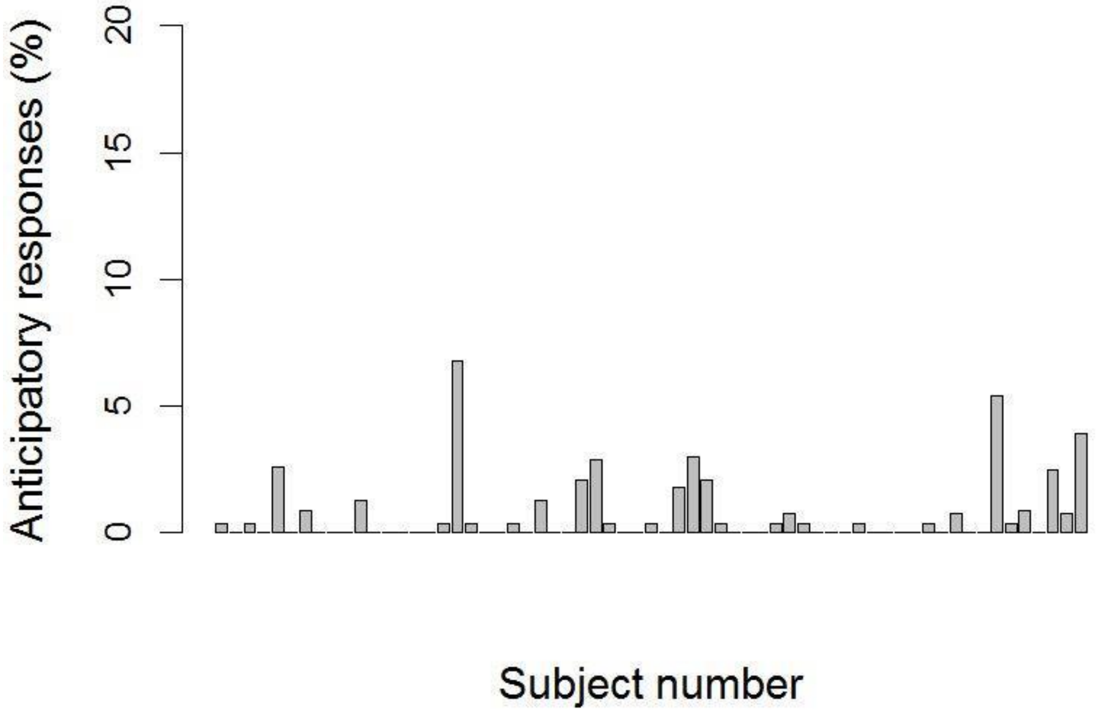
Anticipatory responses (%)
0
5
10
15
20
Subject number

SART RESPONSE VARIABLES: SELF-CAUGHT VERSION

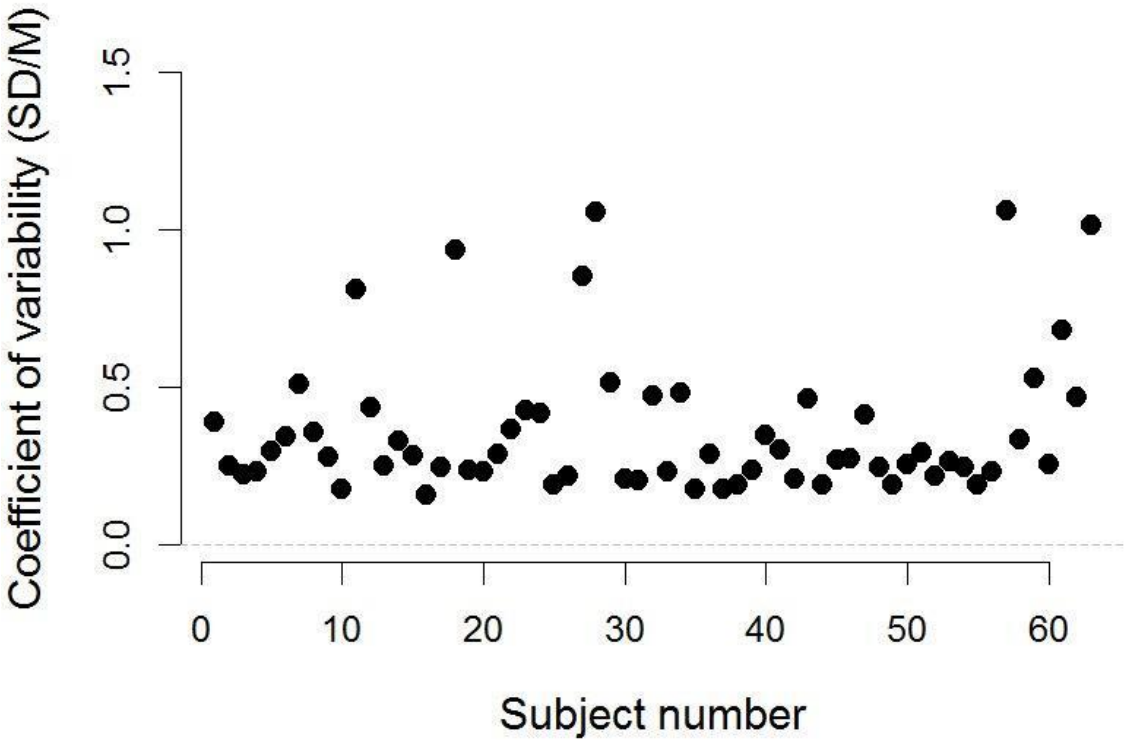

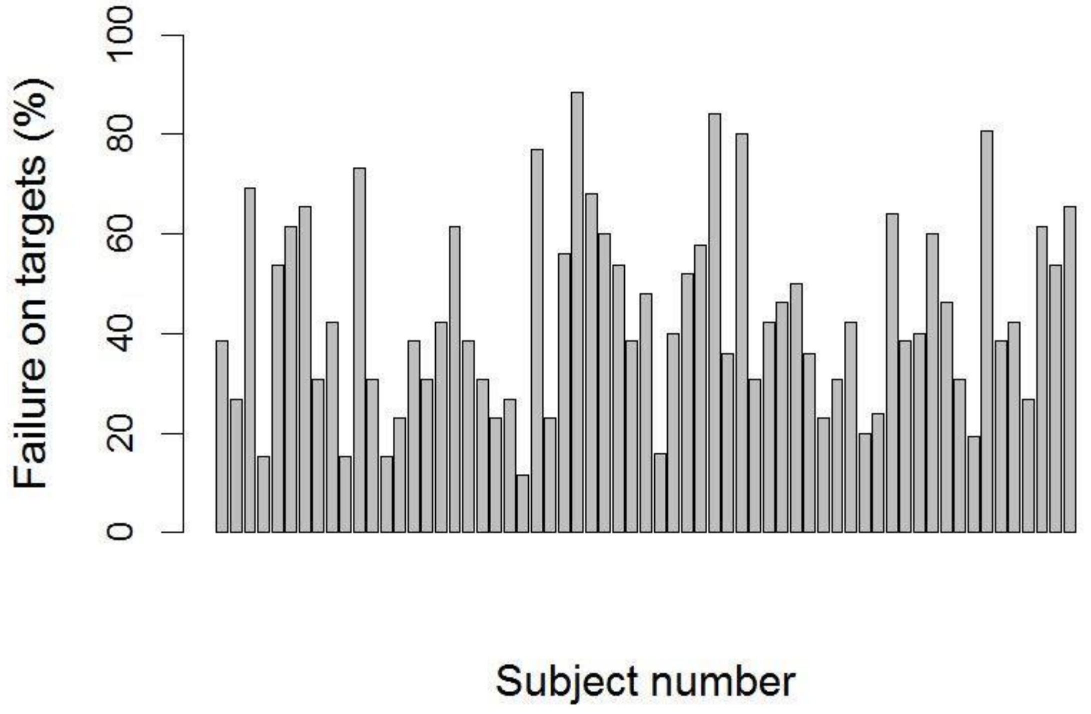

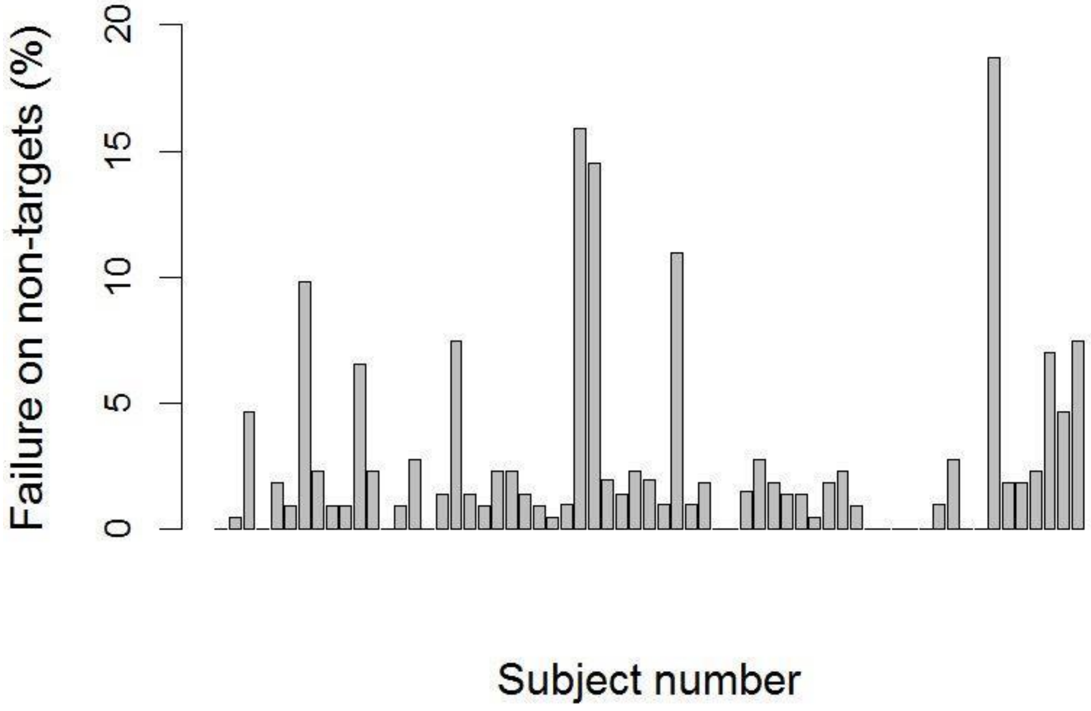
Failure on non-targets (%)
0
5
10
15
20
Subject number

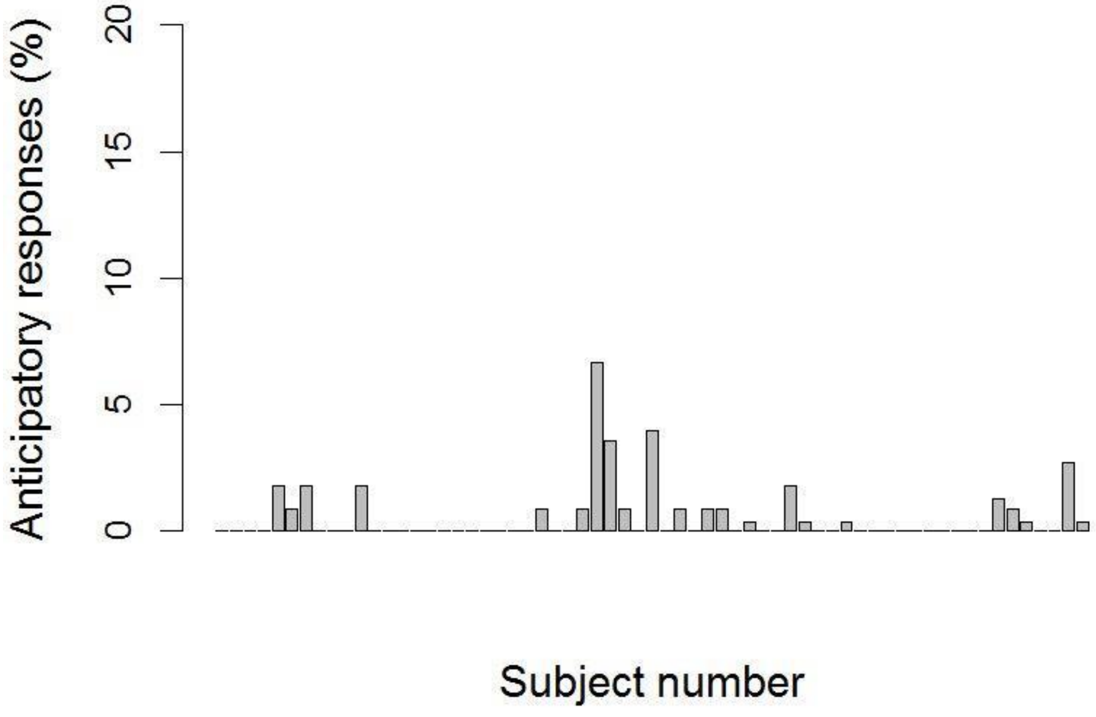
Anticipatory responses (%)
0
5
10
15
20
Subject number

MIND WANDERING DETECTION INDEX: PROBE-CAUGHT

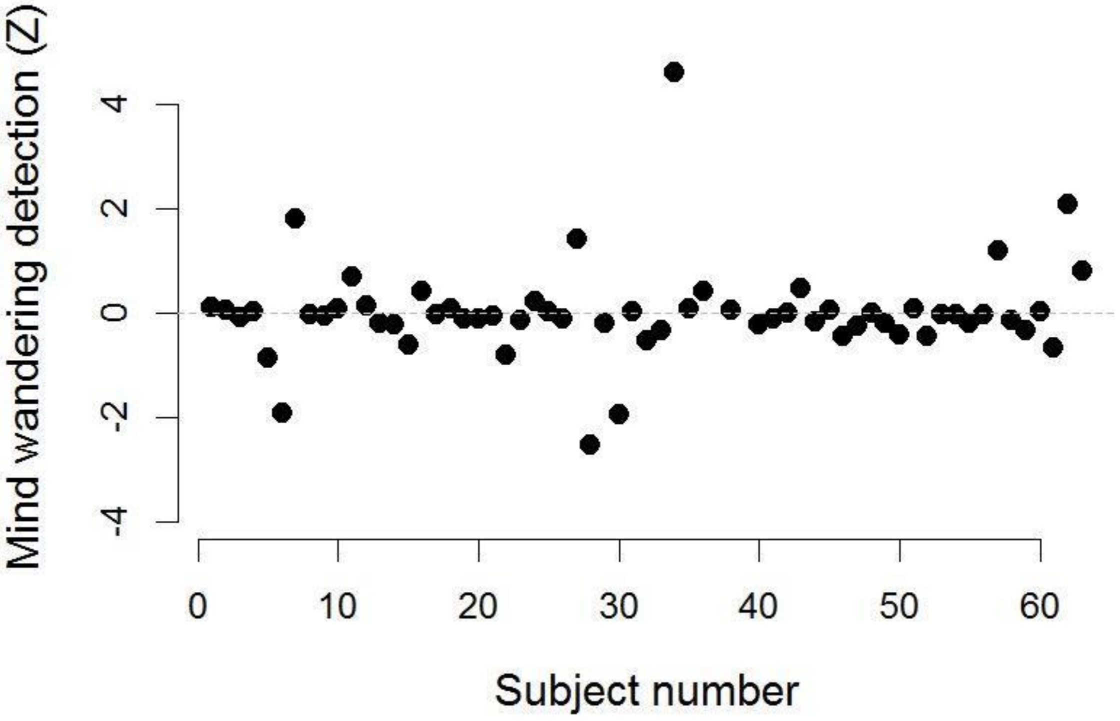

MIND WANDERING DETECTION INDEX: SELF-CAUGHT

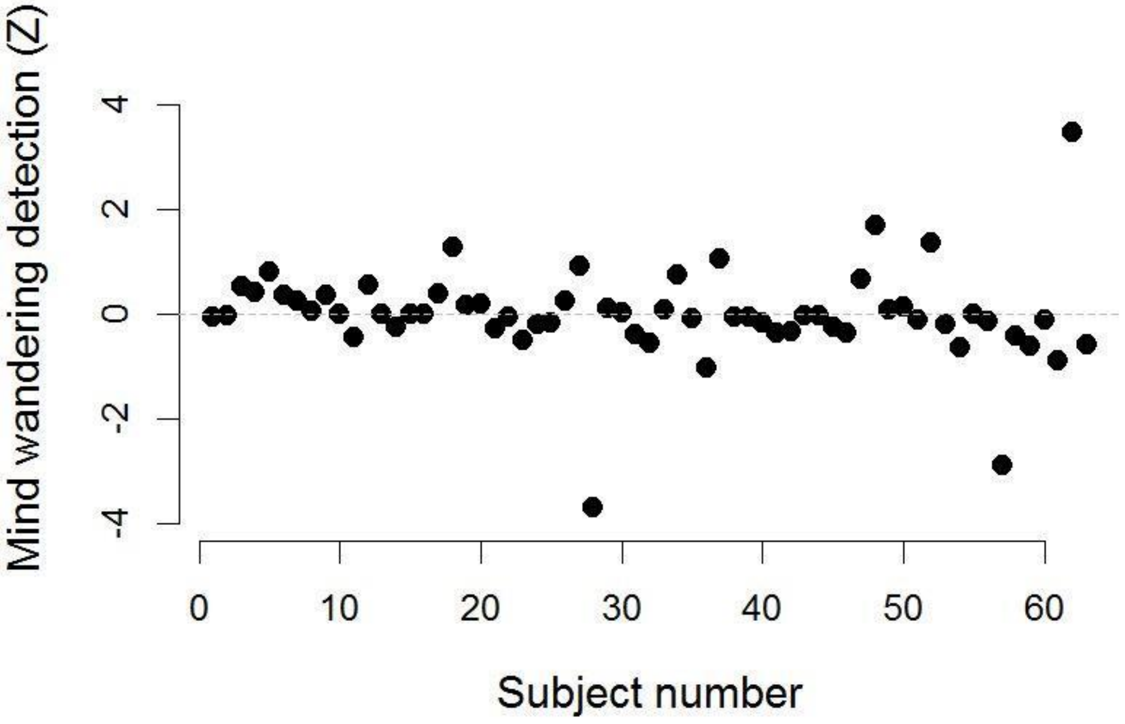

INDEX OF METACOGNITIVE EFFICIENCY

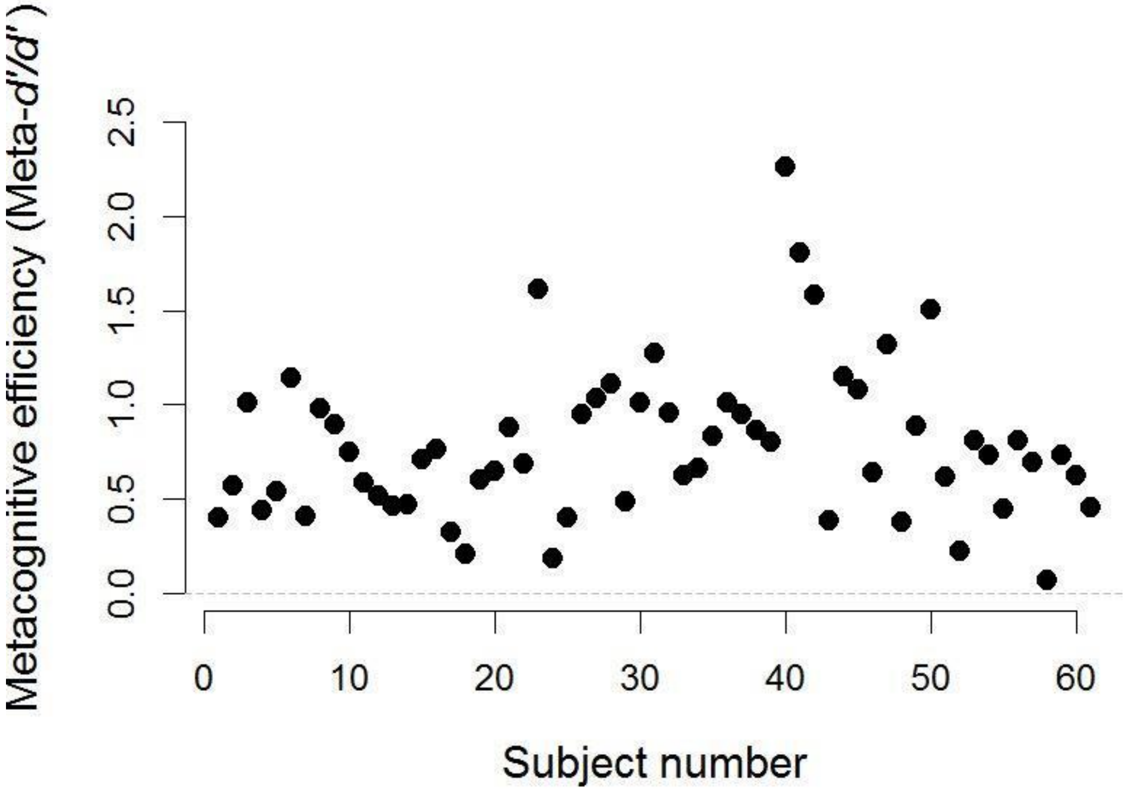

INDEX OF COGNITIVE CONTROL

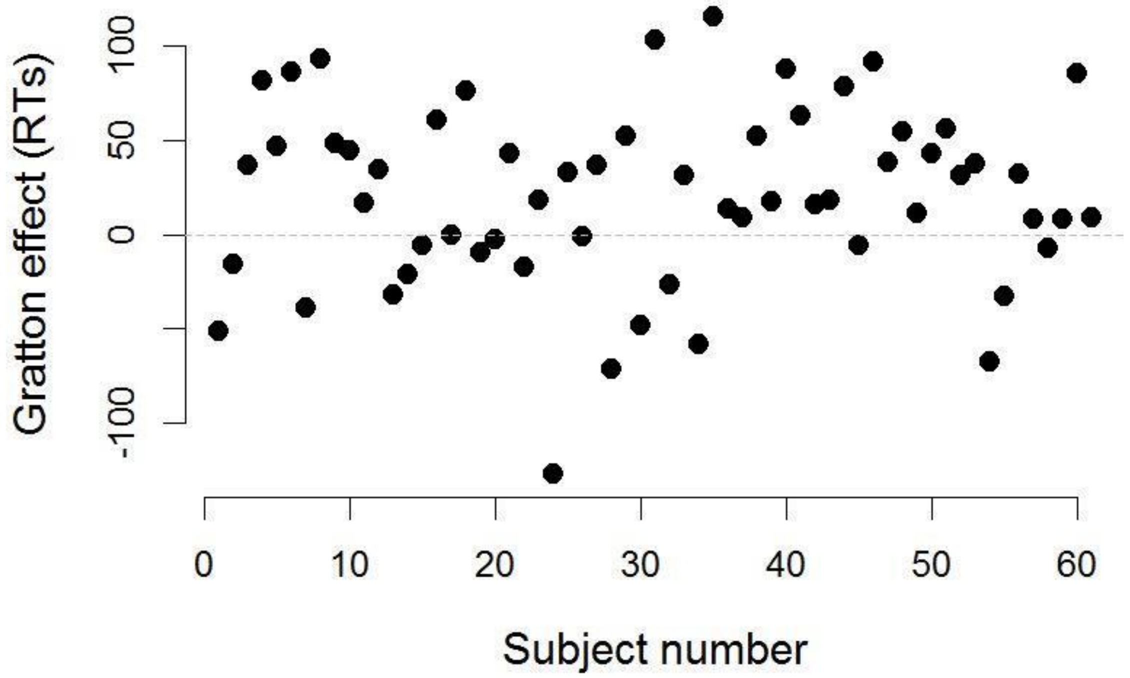

Supplement: S1 File — (PDF) [file pone.0191639.s001.pdf]
