# Supplementary material for: Absence without leave or leave without absence: Examining the interrelations among mind wandering, metacognition and cognitive control
Source: PLoS One. 2018 Feb 9;13(2):e0191639. doi: 10.1371/journal.pone.0191639 (PMC5807058; doi:10.1371/journal.pone.0191639)
Supplement: S1 Text — (DOCX) [file pone.0191639.s002.docx]

**S1 Text.**

**Comparison of the outcome values of the SEMs**

In the following, we compare the SEM model fit of the three different tested methods: Robust regression with the data of all participants, linear regression with the data of all participants, and linear regression after excluding outliers. Robust regression, as reported in the manuscript, resulted in good fit values in the probe-caught version, *χ^2^* (12) = 12.98, *p* = .37, CFI = .97, RMSEA = .037, RMSEA confidence interval = (.000; .124), and in the self-caught version, *χ^2^* (12) = 13.49, *p* = .33, CFI = .96, RMSEA = .044, RMSEA confidence interval = (.000; .126). Using standard linear regression with all data lead to a considerably worsened model fit in the probe-caught version, *χ^2^* (12) = 18.39, *p* = .11, CFI = .93, RMSEA = .093, RMSEA confidence interval = (.000; .174), and in the self-caught version, *χ^2^* (12) = 19.26, *p* = .08, CFI = .94, RMSEA = .098, RMSEA confidence interval = (.000; .176). Finally, removing outliers from the data (detected using the mvoutlier package in R) and then calculating the SEMs with linear regression severely reduced statistical power, especially in the probe-caught version. Although in the probe-caught version (*n* = 37) a close model fit was obtained, *χ^2^* (12) = 8.59, *p* = .74, CFI = 1.00, RMSEA = .000, RMSEA confidence interval = (.000; .123), in the self-caught version (*n* = 50), the model did not have a good fit, *χ^2^* (12) = 19.48, *p* = .08, CFI = .73, RMSEA = .111, RMSEA confidence interval = (.000; .198). In summary, robust regression resulted in superior model fit compared to the two other approaches.

According to MacCallum, Browne and Sugawara (1996), the RMSEA coefficient is indicative of a close fit if its value is below .05, a fair fit if its value is between .05 and .08, and a mediocre fit with a value of .08 to .10. The authors also state that when the upper bound of the confidence intervals of the RMSEA is above .05, the hypothesis test between close fit or non-close fit cannot be decided. Neither of the abovementioned RMSEA confidence intervals is decisive, however, the robust regression RMSEA confidence intervals have a lower upper bound than the standard regression models with outliers.

In sum, it can be concluded that robust regression provided the preferred technique to analyse these data. Nonetheless, we also report the regression results for standard linear regression both with and without outliers, in order to provide the reader with comprehensive information.

***Linear regression with all data.*** Metacognitive efficiency was still positively related to cognitive control, both in the probe-caught version, *β* = .30, *p* = .02, and in the self-caught version, *β* = .29, *p* = .02. The negative relation between metacognitive efficiency and mind wandering detection was absent when using linear regression, both in the probe-caught version, *β* = ‒.17, *p* = .17, and in the self-caught version, *β* = ‒.05, *p* = .67. Metacognitive efficiency was unrelated to the presence of behavioral mind wandering, both in the probe-caught version, *β* = .06, *p* = .65, and in the self-caught version, *β* = ‒.10, *p* = .48. Likewise, cognitive control showed no relation with behavioral mind wandering, in the probe-caught version, *β* = ‒.23, *p* = .10, and in the self-caught version, *β* = ‒.10, *p* = .46. Finally, the covariance between behavioral mind wandering and mind wandering detection had a differential linear tendency in both versions, which did not reach significance in the probe-caught condition, *β* = .22, *p* = .12, but was significant in the self-caught condition, *β* = ‒.34, *p* = .02.

***Linear regression without outliers.*** Metacognitive efficiency was still positively related to cognitive control, both in the probe-caught version, *β* = .35, *p* = .02, and in the self-caught version, *β* = .32, *p* = .02. The negative relation between metacognitive efficiency and mind wandering detection was absent in this SEM calculation, both in the probe-caught version, *β* = ‒.17, *p* = .30, and in the self-caught version, *β* = .01, *p* = .93. Metacognitive efficiency was unrelated to the presence of behavioral mind wandering, both in the probe-caught version, *β* = ‒.01, *p* = .98, and in the self-caught version, *β* = ‒.15, *p* = .39. Likewise, cognitive control showed no relation with behavioral mind wandering, in the probe-caught version, *β* = ‒.28, *p* = .12, and in the self-caught version, *β* = ‒.01, *p* = .94. Finally, the covariance between behavioral mind wandering and mind wandering detection also had a different linear tendency in both versions, and was significant in the probe-caught condition, *β* = .77, *p* = .06, but nonsignificant in the self-caught condition, *β* = ‒.22, *p* = .24.
